# *Artemisia gmelinii* Attenuates Lung Inflammation by Suppressing the NF-κB/MAPK Pathway

**DOI:** 10.3390/antiox11030568

**Published:** 2022-03-16

**Authors:** Seung Yong Kim, Dong-Uk Shin, Ji-Eun Eom, Sun Young Jung, Hyeon-Ji Song, Kyung min Lim, Gun-Dong Kim, Soon-Il Yun, Mi-Yeon Kim, Hee Soon Shin, So-Young Lee

**Affiliations:** 1 Department of Food Science and Technology, Jeonbuk National University, Jeonju 54896, Korea; tmddyd0828@gmail.com (S.Y.K.); songhyeonji@kfri.re.kr (H.-J.S.); siyun@jbnu.ac.kr (S.-I.Y.); 2 Division of Nutrition and Metabolism Research, Korea Food Research Institute (KFRI), Wanju 55365, Korea; 50010@kfri.re.kr (D.-U.S.); 50014@kfri.re.kr (S.Y.J.); limkyungmin@kfri.re.kr (K.m.L.); kgd@kfri.re.kr (G.-D.K.); 3 Department of Food Biotechnology, Korea University of Science and Technology (UST), Daejeon 34113, Korea; 4 Food Functional Evaluation Support Team, Korea Food Research Institute (KFRI), Wanju 55365, Korea; jeeom@kfri.re.kr; 5 Department of Agricultural Convergence Technology, Jeonbuk National University, Jeonju 54896, Korea; 6 Natural F&P Corp., Yongin 16827, Korea; okkite@naturalfnp.com

**Keywords:** cigarette smoke, chronic obstructive pulmonary diseases, lung inflammation, alveolar macrophages, NF-κB pathway

## Abstract

Cigarette smoke (CS) is the main cause of chronic obstructive pulmonary disease (COPD), and continuous CS exposure causes lung inflammation and deterioration. To investigate the protective effects of *Artemisia gmelinii* against lung inflammation in this study, cigarette smoke extract (CSE)/lipopolysaccharide (LPS)-treated alveolar macrophages (AMs) and mice stimulated with CSE/porcine pancreas elastase (PPE) were used. *Artemisia gmelinii* ethanol extract (AGE) was effective in decreasing the levels of cytokines, chemokine, inducible nitric oxide synthase, and cyclooxygenase-2 by inhibiting mitogen-activated protein (MAP) kinases/nuclear factor kappa-light-chain-enhancer of activated B cells (NF-κB) signaling pathway in AMs. Additionally, oral administration of AGE suppressed inflammatory cells’ infiltration and secretion of inflammatory cytokines, chemokines, matrix metallopeptidase 9, and neutrophil extracellular traps in bronchoalveolar lavage fluid from the COPD model. Moreover, the obstruction of small airways, the destruction of the lung parenchyma, and expression of IL-6, TNF-α, IL-1β, and MIP-2 were suppressed by inhibiting NF-κB activation in the lung tissues of the AGE group. These effects are associated with scopolin, chlorogenic acid, hyperoside, 3,4-di-*O*-caffeoylquinic acid, 3,5-di-*O*-caffeoylquinic acid, and 4,5-di-*O*-caffeoylquinic acid, which are the main components of AGE. These data demonstrate the mitigation effect of AGE on lung inflammation via inhibition of MAPK and NF-κB pathways, suggesting that AGE may be instrumental in improving respiratory and lung health.

## 1. Introduction

In recent years, the number of people suffering from respiratory diseases has been increasing gradually because of increasing air pollution, fine dust, and cigarette smoke (CS) [1,2,3]. Among respiratory diseases, chronic obstructive pulmonary disease (COPD) is the third leading cause of death worldwide [4]. The Burden of Obstructive Lung Disease (BOLD) report predicted that approximately 4.5 million people will die due to COPD by 2030 [4]. COPD is a group of lung diseases, including chronic bronchitis and emphysema, which is characterized by irreversible airflow obstruction in small airways, mucus hypersecretion, alveolar wall destruction, and proliferation of airway smooth muscle cells [5]. Patients with COPD have symptoms such as chronic cough, wheezing, phlegm, chest tightness, and shortness of breath [6]. Smoking is the major causative factor in the development of COPD, and >80% of patients with COPD are smokers. CS contains a variety of chemical substances, such as high concentrations of oxidant molecules and unstable free radicals, which induce the formation of reactive oxygen species (ROS), such as hydrogen peroxide, hydroxyl radicals, and superoxide, [7]. Oxidative stress induced by the excessive production of ROS refers to an imbalance between free radicals and antioxidants. This induces lung damage and results in lung diseases such as COPD [8]. CS-induced oxidative stress can cause the secretion of inflammatory mediators, such as chemokine ligand-1 (CXCL-1), CXCL-8, and tumor necrosis factor-α (TNF-α) from alveolar epithelial cells as the first responders to CS [9]. These chemokines bind to CXC chemokine receptor-2 to recruit neutrophils to the airway. Neutrophils recruited to the lung secrete proteolytic enzyme, ROS, matrix metalloproteinase-9 (MMP-9), and MMP-12, leading to mucus hypersecretion and emphysema [10,11]. Furthermore, CS induces the activation of mitogen-activated protein kinases (MAPK) and nuclear factor kappa-light-chain-enhancer of activated B cell (NF-κB) signaling pathways in macrophages, resulting in the secretion of pro-inflammatory cytokines and various inflammatory mediators, leading to lung inflammation and alveolar wall destruction [12]. Recent studies have shown that CS is associated with the infiltration of inflammatory cells such as dendritic cells, macrophages and neutrophils [13,14]. Concordant with these reports, a recent study has shown that the infiltration of macrophages and increased neutrophils in the bronchoalveolar lavage fluid (BALF) and sputum of COPD patients with a history of smoking [15]. Therefore, suppression of the immoderate immune response by CS-stimulated macrophages and neutrophils may be targeted in COPD treatment. Among the many treatment methods, most only focus on the improvement of symptoms and prevention of complications. Additionally, some medications pose a risk of side effects [16]. To overcome this problem, many scientists have been interested in the development of natural products with fewer side effects [17,18]. In this study, we investigated the preventive potential of *Artemisia gmelinii* (AG), which has been widely used for medicinal purposes in Korea.

*Artemisia gmelinii* Weber ex Stechm (AG) belongs to the genus *Artemisia*. AG is widely distributed in Korea, Japan, Sakhalin, Siberia, and China. In Korea, the stem and leaf of AG have been used for medicinal purposes since the Goryeo dynasty, and it has been produced as a drink, yeot, and pill [19]. In a mast cell-mediated model of allergy, *A. iwayomogii* decreased the release of histamine and the production of inflammatory cytokines, such as those reflected by *TNFα* and *IL6* expression, and inhibited the activation of p38 and NF-κB signaling pathways [20]. However, the protective or therapeutic effects of AG against lung inflammation have not yet been demonstrated in COPD models. Therefore, we investigated the attenuating effects of *Artemisia gmelinii* ethanol extract (AGE) on lung inflammation by suppressing NF-kB/MAPK activation in a CS extract (CSE)/lipopolysaccharide (LPS)-stimulated alveolar macrophage cell line (MH-S) and CSE/porcine pancreatic elastase (PPE)-induced mouse model of COPD.

## 2. Materials and Methods

### 2.1. Materials

RPMI 1640, fetal bovine serum (FBS), penicillin–streptomycin, and Dulbecco’s phosphate-buffered saline (DPBS) were purchased from Welgene (Gyeongsan-si, Korea), and LPS, 4′,6-diamidine-2′-phenylindole dihydrochloride (DAPI), dexamethasone, and β-mercaptoethanol (2-ME) were purchased from Sigma-Aldrich (Saint Louis, MO, USA). WST-1 solution was obtained from Cellvia (Seoul, Korea). A nitric oxide (NO) assay kit and NE-PER™ Nuclear and Cytoplasmic Extraction Reagent were obtained from Thermo Fisher Scientific (Waltham, MA, USA). IL-6 and TNF-α enzyme-linked immunosorbent assay (ELISA) kits were purchased from BD Biosciences (San Jose, CA, USA) and MIP-2 and IL-1β ELISA kits were purchased from R&D Systems (Minneapolis, MN, USA). We purchased 10× cell lysis buffer, p-ERK, ERK, p-JNK, JNK, p-p38, p38, IκBα, NF-κB p65, COX-2, iNOS, lamin B1, HO-1, NFR-2 and anti-mouse or rabbit IgG antibodies, and anti-rabbit IgG (H + L), F(ab’)2 fragment from Cell Signaling Technology (Danvers, MA, USA). p-38 inhibitor SB203580 and ammonium pyrrolidine dithiocarbamate (PDTC) were purchased from Sigma (St. Louis, MO, USA). β-actin antibodies were purchased from Santa Cruz Biotechnology (Houston, TX, USA). For Western blotting, Mini-PROTEAN^®^ TGX™ Precast Gels, 10× Tris/Glycine/SDS buffer, and a DC™ protein assay kit were obtained from Bio-Rad (Hercules, CA, USA), and the lung tissue dissociation kit was obtained from Miltenyi Biotec (Cologne, Germany). For quantitative reverse-transcription polymerase chain reaction (qRT-PCR), Qiazol and QIAzol^®^ Lysis Reagent were purchased from Qiagen (Valencia, CA, USA). *IL6*, *TNFA*, *MIP2*, and *IL1B* primers were purchased from Chancebio (Jeonju-si, Korea). PPE was obtained from Merck-Millipore (Burlington, MA, USA) and was used to induce lung emphysema in mice. All the other chemicals used were of the highest commercially available grade.

### 2.2. Preparation of Artemisia Gmelinii Ethanol Extract (AGE)

AGE used in this study was provided by Natural F&P Co. Ltd. (Cheongju-si, Korea). Briefly, AG herbs were collected from Jecheon-si (Chungcheong-do, Korea) and Youngcheon (Gangwon, Korea). Raw AG was dried, powdered, and then extracted with 15-times volumes of 50% ethanol by heating (±50 °C) for 6 h. AGE was subsequently filtered using a cartridge filter. Using a Rotavapor R-210 (BÜCHI Labortechnik AG, Flawil, Switzerland), the filtered extract was concentrated under vacuum at 50 °C and then dried. AGE was dissolved in dimethyl sulfoxide prior to use in the experiments.

### 2.3. Preparation of Cigarette Smoke Extract (CSE)

Research-grade cigarettes 3R4F were obtained from the University of Kentucky (Lexington, KT, USA) to prepare CSE. For CSE preparation, filter-removed cigarettes were burned, and the smoke passed through 10 mL of PBS using a vacuum pump for 1 min. After filtering through a Millex-LCR hydrophilic PTFE 0.45-μm filter (Merck-Millipore, Burlington, MA, USA), the absorbance of the filtered CSE at 320 nm was measured using an Epoch microplate reader (BioTek, Winooski, VT, USA) and adjusted to an optical density value in the range of 0.9–1.0 for standardization of the CSE. CSE was stored at −80 °C until use in the experiment.

### 2.4. Cell Culture

MH-S mouse alveolar macrophages (AMs) were obtained from ATCC (Manassas, VA, USA). MH-S macrophages were cultured in RPMI-1640 medium containing 10% (*v*/*v*) FBS, 1% (*v*/*v*) penicillin streptomycin and 0.05 M 2-ME. Cells were incubated at 37 °C in a humidified atmosphere containing 5% CO_2_ and 95% air for 24 h.

### 2.5. Animal Study

Male BALB/c mice (5-weeks-old, 18–20 g) were purchased from Orient Bio (Gyeonggi, Korea). After a 1-week acclimation period, mice were randomly divided into the following six groups: the control group (Naïve, *n* = 10), the CSE/PPE-treated group (COPD, *n* = 15), CSE/PPE and 50 mg/kg AGE-treated group (AGE 50, *n* = 10), CSE/PPE and 100 mg/kg AGE-treated group (AGE 100, *n* = 10), CSE/PPE and 200 mg/kg AGE-treated group (AGE 200, *n* = 10), and CSE/PPE and 2.5 mg/kg dexamethasone-treated group (DEX, *n* = 10), as the positive control. To induce COPD, mice were intranasally treated with 20 μL of CSE (100%) and PPE (1.8 U/Head) from days 7 to 35, three times a week, and once every 2 weeks, respectively. The COPD group and 50, 100, and 200 AGE groups were orally administered 200 μL/mouse of PBS and AGE diluted in PBS from days 0 to 35. In the DEX group, 200 μL/mouse of dexamethasone diluted in PBS was orally administered from days 7 to 35, three times a week, to consider side effects such as body weight loss, insomnia, and depression [21,22], before treatment with CSE and PPE. The mice were sacrificed on day 36. Blood, lung tissue, and BALF were collected for further analyses. All animal procedures were carried out in accordance with the guidelines for animal use and care of the Korea Food Research Institute (approval number:KFRI-M-20028) Animals were maintained in the same room under conventional conditions, with a regular 12 h light/dark cycle, and temperature and relative humidity maintained at 23 ± 2 °C and 50 ± 5%, respectively. Mice were allowed free access to food and water.

### 2.6. Cell Viability

A WST-1 assay was performed to evaluate the effects of AGE on cell cytotoxicity. MH-S macrophages (5 × 10^5^ cells/mL) were seeded into 48-well plates and were then treated with different doses of AGE (25, 50, and 100 µg/mL), with or without CSE (1%)/LPS (10 ng/mL) at 37 °C for 24 h. The next day, the supernatants were collected and used to measure other factors. WST-1 solution was mixed with the medium at a ratio (1:10). Then, 300 μL of the mixed solution was added to each well from which the supernatants had been removed, followed by incubation at 37 °C for 30 min. Absorbance at 450 nm was measured using an Epoch microplate reader (BioTek, Winooski, VT, USA).

### 2.7. Measurement of Nitric Oxide (NO) Production in MH-S Macrophages

To measure the level of NO production, MH-S macrophages (5 × 10^5^ cells/mL) in 48-well plates were co-treated with CSE (1%)/LPS (10 ng/mL) and AG (25, 50 and 100 µg/mL) at 37 °C for 24 h. The next day, the supernatants were collected and used to measure the level of NO production by using Nitric Oxide assay kit. Briefly, 50 µL of standards and supernatants was added into each well. Then, 50 µL of Griess Reagent I and II were also added and the plate was incubated at room temperature for 10 min. The absorbance of each well was measured at 540 nm by Epoch microplate reader (BioTek, Winooski, VT, USA).

### 2.8. Measurement of Cytokines and Chemokines Production in MH-S Macrophages and BALF

To measure the level of IL-6 and MIP-2, MH-S macrophages (5 × 10^5^ cells/mL) in 48-well plates were co-treated with CSE/LPS and AGE as above. The following day, the supernatants were collected and used to measure IL-6 and MIP-2 levels using an ELISA kit. Briefly, each well was coated overnight with the capture antibody. The next day, each well was washed with washing buffer and blocked by adding blocking solution for 1 h. Standards and supernatants were diluted with the reagent diluent and were added to the appropriate wells for 2 h. Detection antibody and horseradish peroxidase (HRP) mixed with reagent diluent were added to the appropriate wells for 1 h. The substrate solution was added to the appropriate wells for 30 min. To end the reaction, stopping solution was added to each well. The absorbance of each well was measured at 450 nm using an Epoch microplate reader (BioTek). IL-6, MIP-2, and MMP-9 levels in BALF were quantified by ELISA.

Chemokines, such as eotaxin, keratinocyte chemoattractant, MCP-1, MDC, RANTES, and TARC, in the BALF were assessed using a Q-plex assay kit (Quansys Biosciences, Logan, UT, USA). Briefly, BALF samples and standard solutions for the calibration curve diluted 1:2 with sample diluent were added to a Q-Plex™ Array 96-well plate and were shaken for 1 h at room temperature. After washing, the detection mixture was added to the plate for 1 h at room temperature, followed by shaking. Streptavidin-HRP was added to the plate for 15 min at room temperature, followed by shaking. Substrate solutions were then added to produce a response. In order to measure the intensity of spot response, the image of the plate was captured using the Q-View™ Imager Pro (Quansys Biosciences).

### 2.9. Western Blot Analysis of iNOS&COX-2 and MAPK/NF-ĸB in MH-S Macrophages

MH-S macrophages were seeded in 6-well cell culture plates and incubated at 37 °C for 24 h. The following day, MH-S macrophages were treated with AGE (25, 50, and 100 µg/mL) for 2 h prior to a stimulation period with CSE (1%)/LPS (10 ng/mL). To extract the protein, MH-S macrophages were collected in DPBS and lysed in an ice-cold cell lysis buffer with protease inhibitor. The concentration of extracted proteins in each sample was measured using the Bradford protein assay. In total, 40 micrograms of the protein mixture from each sample was loaded into the wells of Mini-PROTEAN^®^ TGX™ Precast Gels. After electrophoresis, the proteins in the gels were transferred onto Western blotting filter membranes for 50 min at 25 V, using a Pierce G2 Fast Blotter (Thermo Fisher Scientific). After the transfer, the membranes were blocked with 5% skimmed milk in PBS-Tween 20 (PBST) for 30 min at room temperature. Then, the membranes were incubated with diluted p-ERK, ERK, p-JNK, JNK, p-p38, p38, IκBα, NF-κB p65, COX-2, iNOS, lamin B1, and β-actin antibodies overnight at 4 °C. The next day, the membranes were washed three times with PBST for 5 min. After washing, the membranes were incubated with anti-mouse or rabbit IgG antibodies conjugated with HRP for 1 h at room temperature and washed three times with PBST for 5 min. The protein bands were quantified using a Fusion Solo S (Vilber, Lamirault, France).

### 2.10. Immunofluorescence Assay of Nuclear Translocation of NF-κB in MH-S Macrophages

MH-S macrophages (2 × 10^5^ cells/mL) were seeded onto 8-well chamber slides. After 24 h, MH-S macrophages were treated with AGE (100 μg/mL) for 2 h prior to a stimulation period with CSE (1%)/LPS (10 ng/mL). Macrophages were fixed with 4% formaldehyde for 15 min and permeabilized with 0.2% Triton X-100 for 5 min at room temperature. Macrophages were blocked with 1% bovine serum albumin and incubated with diluted anti-NF-κB p65 antibody overnight at 4 °C. The next day, the macrophages were washed three times and stained with anti-rabbit IgG (H + L) and F(ab’)2 fragment. After 30 min, macrophages were washed with DPBS and distilled water. After drying the wells, DAPI solution was added to the macrophages. Microscopy was performed at 408 nm (46-diamidino-2-phenylindole) and 488 nm (fluorescein isothiocyanate) using an Eclipse Ti confocal fluorescence microscope (Nikon, Tokyo, Japan). Fluorescence intensity was measured using ImageJ software.

### 2.11. RNA Isolation and Real-Time Quantitative Polymerase Chain Reaction (RT-PCR) in MH-S Macrophages and Lung Tissues

MH-S macrophages (5 × 10^5^ cells/mL) were seeded in 12-well plates and incubated at 37 °C for 24 h. After 24 h, MH-S macrophages were treated with AGE (25, 50, and 100 μg/mL) for 2 h prior to a stimulation period with CSE (1%)/LPS (10 ng/mL). Total RNA was extracted from MH-S macrophages using the QIAzol^®^ Lysis Reagent. On the other hand, to extract mRNA from the lung tissues of mice, the isolated left lungs were placed into a 2-mL tube, to which QIAzol^®^ Lysis Reagent and beads were added. The tissue was then homogenized using a TissueLyser II (Qiagen) at 30 Hz for 1 min. The extracted RNA was quantified using a Nanodrop 2000 spectrophotometer (Thermo Fisher Scientific). Extracted RNA was used to synthesize cDNA at 45 °C for 1 h, followed by 95 °C for 5 min, in a C1000™ Thermal Cycler (Bio-Rad). The solutions for qRT-PCR contained 5.5 μL RNase free water, 2.5 μL of each primer, 2 μL of cDNA template and 12.5 μL of SYBR Green PCR mix in a final reaction volume of 25 μL. The qRT-PCR cycling conditions were 95 °C for 10 min, and 50 cycles each consisting of 95 °C for 30 s, 60 °C for 25 s, and 72 °C for 25 s using a Rotor-Gene Q (Qiagen). The sequences of primers used in this study are as follows: TNF-α forward, 5′-TCTTCTCATTCCTGCTTGTGG-3′ and reverse, 5′- GGTCTGGGGCATAGAACTGA-3′; IL-1β forward, 5′-ACCTGGGCTGTCCTGATGAGAG-3′ and reverse, 5′-GTTGATGTGCTGCTGCGAGAT-3′; IL-6 forward, 5′-TGGGACTGATGCTGGTGACAAC-3′ and reverse, 5′- AGCCTCCGACTTGTG AAGTGGT-3′; MIP-2 forward, 5′-CGGCAATGAAGCTTCTGTAT-3′ and reverse, 5′-CCTTGAAAC TCTTTGCCTCA-3′; iNOS forward, 5′- CAGCGGAGTGACGGCAAACAT-3′ and reverse, 5′-GCAAGACCAGAGGCAGCACATC-3′; COX-2 forward, 5′-CTGGTGCCTGGTCTGAT GATGTATG-3′ and reverse, 5′-TCTCCTATGAGTATGATCTGCTGGTT-3′; β-actin used as housekeeping gene: forward, 5′-GCTCAGTAACAGTCCGCCTAGA-3′ and reverse, 5′-TGTCCACCTTCCAGCAGATGT-3′.

### 2.12. Analysis of the Differential Cell Counts in BALF

To measure the total cell count in BALF, the lungs were lavaged with DPBS to collect BALF. After injecting 1 mL PBS into the lungs through the mouse airway, approximately 700 μL of BALF was obtained. BALF (10 µL) was mixed with an equal volume of Accustain T solution (NanoEntek, Seoul, Korea). Then, we loaded 12 μL of the mixed solution into the Accuchip channel and counted the total number of cells using the ADAM-MC™ Automated Cell Counter (NanoEntek). The BALF samples obtained were centrifuged (300× *g*, 5 min) to separate supernatants and cell pellets. The cell pellets were resuspended in 700 μL of PBS. After centrifugation (1000 rpm, 10 min, 4 °C) using a Centrifuge 5403 Cytospin device (Eppendorf, Hamburg, Germany), 150 μL of resuspended solution was placed onto slides. Then, the immune cells in the slides were stained following the protocol of the Diff-Quick staining reagent (38721; SYSMEX, Kobe, Japan), and the number of macrophages and neutrophils were counted using a microscope.

### 2.13. Protein Simple Capillary Immunoassay of Nuclear Translocation of NF-κB in Lung Tissues

To extract proteins from the lung tissues of mice, isolated right lungs were homogenized using a lung tissue dissociation kit (130-095-927, Miltenyi Biotec, Bargisch Gladbach, Germany). After homogenization, the samples were collected in DPBS and were lysed in ice-cold cell lysis buffer with a protease inhibitor. To separate the cytoplasm and nucleus, the NE-PER™ Nuclear and Cytoplasmic Extraction Reagent (Thermo Fisher Scientific) was used. The concentration of extracted proteins in each sample was measured using the Bradford protein assay. The expression of IκBα and β-actin in the cytoplasmic fraction and NF-κB p65 and lamin B1 in the nuclear fraction were measured using the ProteinSimple Wes capillary immunoassay system (Protein Simple, San Jose, CA, USA).

### 2.14. Histological Analysis of Lung Tissues

After the mice were euthanized, the left lung tissue was collected and excised. The samples were immediately fixed in 10% neutral buffered formalin solution for 3 days at room temperature. Then, they were washed for 24 h using tap water and dehydrated in a series of ethanol solutions. Ethanol was removed using xylene, and the tissue was embedded in paraffin. The lung tissues were sectioned at 4.5 μm thickness. To estimate the general structure of the lung tissues, hematoxylin and eosin (H&E) (Sigma-Aldrich, Saint Louis, MO, USA) staining was performed and sections were observed under a microscope.

### 2.15. NETosis Assay

Neutrophil extracellular trap (NET) release was quantified using a SYTOX green dye (Invitrogen, Carlsbad, CA, USA), which is a marker of necrotic cell death that binds nucleic acids when the cell membrane is broken. To test for NETosis, BALF was seeded into a 96-well black plate. After the SYTOX green dye was diluted 1:500 with Hank’s balanced salt solution, it was added to the wells and incubated for 10 min. Fluorescence intensities were measured using SpectraMax i3 (Molecular Devices, San Jose, CA, USA) at 480/530 nm.

### 2.16. High-Performance Liquid Chromatography (HPLC) Analysis

The bioactive compounds in AGE were analyzed using an Agilent 1260 HPLC system (Agilent Technologies, Santa Clara, CA, USA) equipped with an Agilent 1260 TCC (G1316A) UV-vis detector (Agilent Technologies). The mobile phase consisted of acetonitrile (solvent A) and water containing 0.1% formic acid (solvent B), and the HPLC gradient profile was as follows: 14% A at 0–15 min, 14–26% A at 15–18 min, 26% A at 18–40 min, 26–90% at 40–50 min, and 90–14% A at 50–60 min. Five microliters of AGE (30 mg/mL) was injected into a Sunfire C18 column (5 μm, 4.6 × 250 mm; Waters Corp., Milford, MA, USA) at a column temperature of 30 °C. The flow rate was 0.6 mL/min and eluted compounds were detected at 360 nm. As reference compounds used in this study, scopolin and scoparone were obtained from PhytoLab (Vestenvergsgreuth, Germany); hyperoside was obtained from HWI Analytic GmbH (Ruelzheim, Germany); scoplectin, 3,4-di-*O*-caffeolyquinic acid, 3,5-di-*O*-caffeolyquinic acid, and 4,5-dicaffeolyquinic acid were obtained from Sigma-Aldrich (St. Louis, MO, USA). HPLC-grade solvents were obtained from Thermo Fisher Scientific.

### 2.17. Statistical Analysis

All data were analyzed using GraphPad Prism software (version 9.0; La Jolla, CA, USA) and were statistically examined using one-way analysis of variance followed by Bonferroni post hoc test to compare multiple groups using SPSS software version 20 (IBM SPSS Inc., Armonk, NY, USA). The Bonferroni post hoc test was used to robustly control the family-wise error rate increased with increasing the number of comparisons performed between 5 or 7 groups in this study. The values for in vitro data are expressed as the mean ± standard deviation (SD), whereas in vivo data are expressed as the mean ± standard error (SE) of independent experiments. A *p*-value < 0.05 was considered to be statistically significant.

## 3. Results

### 3.1. Effect of AGE on Cell Viability and iNOS and COX-2 Expression in MH-S Macrophages

**Figure 1:**
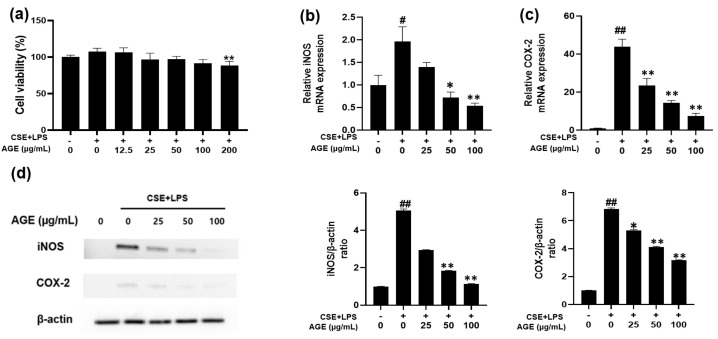
Effect of the *Artemisia gmelinii* ethanol extract (AGE) on cell viability and the expression levels of *NOS2* and *COX2* mRNA and protein in MH-S macrophages. For the cell viability assay (**a**), the MH-S macrophages were co-treated with 12.5, 25, 50, 100, 200 µg/mL of AGE and CSE (1%)/LPS (10 ng/mL) for 24 h. Cell viability was estimated by the WST assay. To determine the expression of *NOS2 COX2* mRNA (**b**,**c**) and protein (**d**), MH-S macrophages were co-treated with 25, 50, 100 µg/mL of AGE and CSE (1%)/LPS (10 ng/mL) for 1 h (*NOS2* mRNA), 6 h (*COX2* mRNA), 12 h (iNOS and COX2 protein), respectively. The expression of mRNA and protein was measured by quantitative real-time polymerase chain reaction and Western blotting, respectively. Western blot data were quantitatively analyzed using FUSION Solo S software. Data are presented as means ± SD (*n* = 3). The significant differences among the groups were analyzed using one-way analysis of variance by Bonferroni’s test (# *p*  <  0.05 and ## *p* < 0.01 compared to the control group; * *p* < 0.05 and ** *p* < 0.01 compared to the CSE + LPS-stimulated group).

Cell viability was tested to determine the non-cytotoxic concentration of AGE. As shown in Figure 1a, cytotoxicity was not observed up to an AGE concentration of 100 μg/mL. Thus, 25, 50, and 100 μg/mL AGE were used in the experiment. After assessing cytotoxicity, iNOS and COX-2 levels were measured to determine the anti-inflammatory ability of AGE. As described in Figure 1b,c, *NOS2* and *COX2* mRNA levels were increased in CSE/LPS-stimulated macrophages. In the AGE-treated macrophages, these levels decreased in a dose-dependent manner. Similarly, the iNOS and COX-2 protein levels were enhanced in CSE/LPS-stimulated macrophages. However, these levels were inhibited in AGE-treated macrophages in a concentration-dependent manner (Figure 1d). These results suggest that AGE inhibits the production of iNOS and COX-2.

### 3.2. Effect of AGE on Pro-Infammatory Cytokines and Chemokines and the NF-κB Pathway in MH-S Machrophages

**Figure 2:**
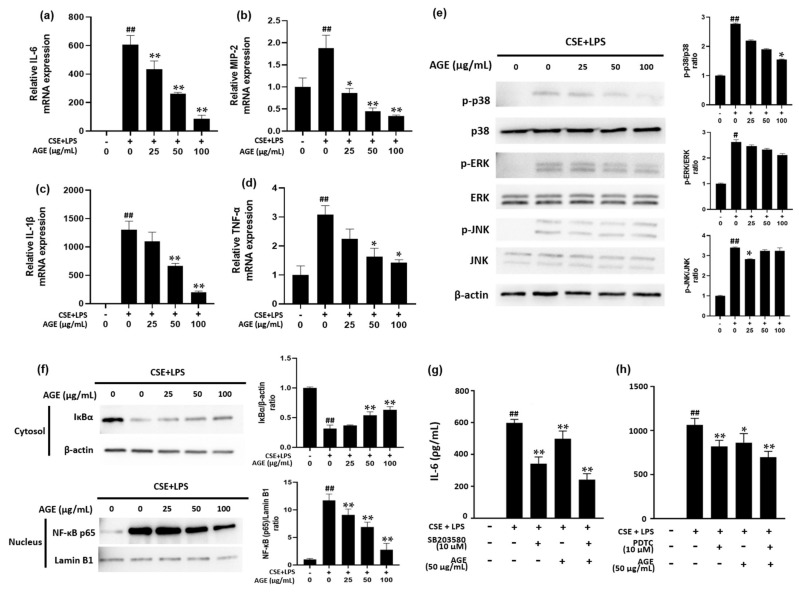
Effect of the *Artemisia gmelinii* ethanol extract (AGE) on the production of cytokines and chemokines and the activation of MAPK/NF-κB pathways in MH-S macrophages. First, to measure mRNA levels of *IL6* (**a**), *MIP2* (**b**), *IL1β* (**c**), and *TNFα* (**d**), MH-S macrophages were pre-treated with 25, 50, 100 µg/mL of AGE for 2 h and were then treated with CSE (1%)/LPS (10 ng/mL) for 3 h. The mRNA levels were analyzed using quantitative real-time polymerase chain reaction. Secondly, to determine the activation of MAPK (**e**)/NF-κB (**f**) pathways, MH-S macrophages were pre-treated with 25, 50, 100 µg/mL of AGE for 2 h and were then treated with CSE (1%)/LPS (10 ng/mL) for 15 and 30 min, respectively. Protein expression of p-p38, p38, p-ERK, ERK, p-JNK, JNK, IκBα, NF-κB p65, β-actin, and lamin B1 was assessed by Western blotting and data were analyzed using FUSION Solo S software. To measure IL-6, the MH-S cells were pre-incubated with SB203580 (for 1 h) or PDTC (30 min) followed by treatment of CSE (1%)/LPS (10 ng/mL) and AGE for 24 h. The protein levels were analyzed for IL-6 (**g**,**h**) by ELISA. Data are presented as means ± SD (*n* = 3). The significant differences among the groups were analyzed using one-way analysis of variance by Bonferroni’s test (# *p*  <  0.05 and ## *p* < 0.01 compared to the control group; * *p* < 0.05 and ** *p* < 0.01 compared to the CSE + LPS-stimulated group).

IL-6, TNF-α, and IL-1β are pro-inflammatory cytokines, while MIP-2 is a chemokine related to the recruitment of neutrophils. As shown in Figure 2, the mRNA levels of IL-6, TNF-α, IL-1β, and MIP-2 in the CSE/LPS-stimulated MH-S macrophages were significantly higher than those in the control group. However, these levels decreased in a dose-dependent manner in AGE-treated macrophages.

The secretion of pro-inflammatory mediators is related to the activation of the MAPK cascade. As shown in Figure 2e, CSE/LPS markedly activated phosphorylation of p38, ERK, and JNK in CSE/LPS-stimulated macrophages. However, treatment with AGE suppressed the phosphorylation of p38 as the concentration of AGE increased. Quantitative data showed that the p-p38/p38 ratio decreased in a dose-dependent manner.

**Figure 3:**
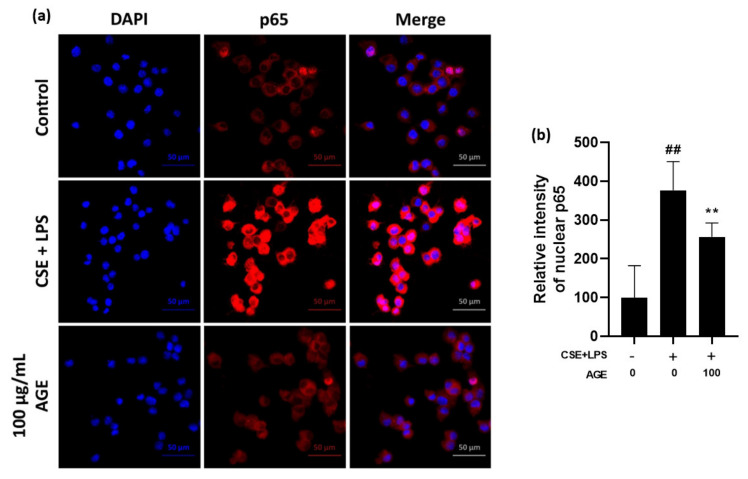
Effect of *Artemisia gmelinii* ethanol extract (AGE) on the activation of NF-κB in MH-S macrophages. (**a**) MH-S macrophages were pre-treated with 25, 50, 100 µg/mL of AGE for 2 h and were then treated with CSE (1%)/LPS (10 ng/mL) for 30 min. NF-κB nuclear translocation was estimated using an anti-p65 antibody and an Alexa Fluor 488-conjugated anti-rabbit IgG antibody. The nuclei were stained with DAPI (scale bar: 50 µm). The images show representative plots of three replicate experimens. (**b**) The relative p65 intensity in the nuclear region. Mean fluorescence intensity of each individual cell nucleus was measured using ImageJ software. The significant differences among the groups were analyzed using one-way analysis of variance by Bonferroni’s test ## *p* < 0.01 compared to the control group; ** *p* < 0.01 compared to the CSE + LPS-stimulated group).

NF-κB and MAPK are involved in the transcriptional induction of pro-inflammatory mediators. In Figure 2f, IκBα in the cytosol was degraded in the CSE/LPS-stimulated MH-S macrophages, whereas IκBα degradation was attenuated in the AGE-treated macrophages in a dose-dependent manner. Quantitative data showed that the IκBα/β-actin ratio was higher in the AGE-treated macrophages than in the CSE/LPS-stimulated MH-S macrophages. In contrast, the expression of NF-κB p65 protein in the nucleus was increased in CSE/LPS-stimulated MH-S macrophages while the NF-κB p65/lamin B1 ratio was decreased in macrophages treated with AGE compared to CSE/LPS stimulated MH-S macrophages. Consistent with the Western blotting (Figure 2f), the increased fluorescence intensity of p65 observed in the nuclei of the CSE/LPS-stimulated MH-S macrophages was significantly reduced by treatment with 100 μg/mL of AGE, as shown in Figure 3a,b.

Next, we investigated whether AGE could inhibit IL-6 expression through the p38 and NF-κB pathway in the CSE/LPS stimulated MH-S cells. We measured the IL-6 after treatment with pharmacological p38 and NF- κB inhibitors, SB203580 and PDTC, in the absence or presence of AGE. As anticipated, the CSE/LPS-induced IL-6 was significantly suppressed by SB203580 and PDTC treatment (Figure 2g,h). AGE also significantly attenuated IL-6 production in CSE/LPS-stimulated MH-S cells, similar to the PDTC treatment. In addition, the combination of AGE and either SB203580 or PDTC treatment significantly inhibited IL-6 production compared to a single treatment of AGE or each inhibitor in the CSE/LPS-stimulated MH-S cells (Figure 2g,h).

Taken together, these results suggest that AGE suppresses the production of pro-inflammatory cytokines and chemokines by blocking the p38 MAPK and NF-κB pathways in MH-S macrophages.

### 3.3. Effect of AGE on Inflammatory Cell Counts, Cytokines, Chemokines, MMP-9, and NETosis in BALF

**Figure 4:**
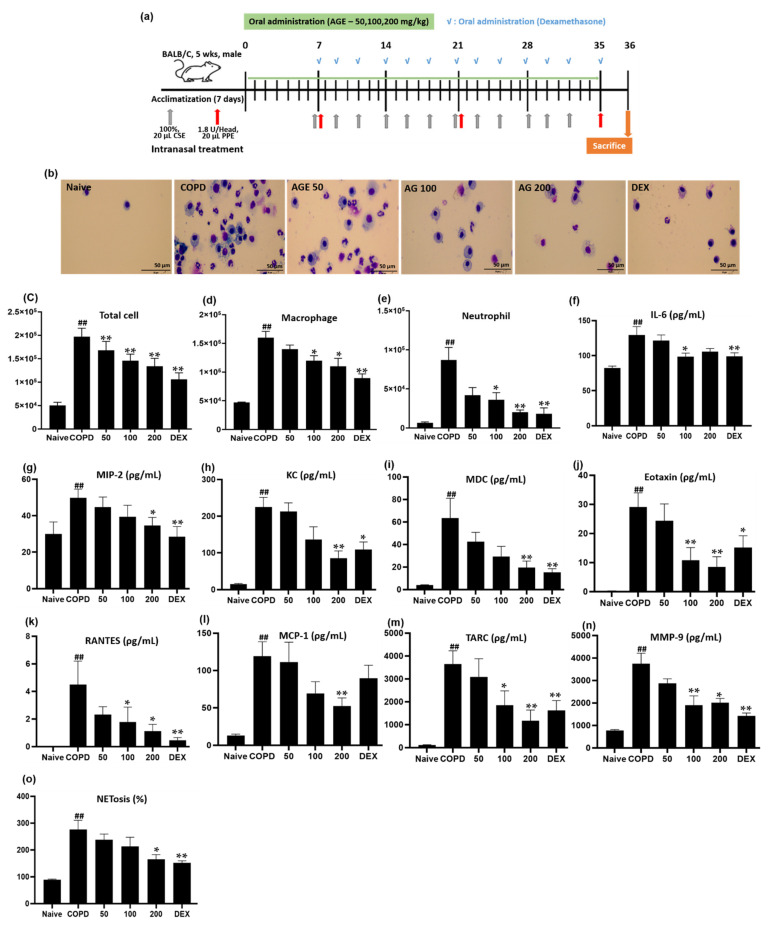
Effect of the *Artemisia gmelinii* ethanol extract (AGE) on the the inflammatory cell count and the production of cytokines and chemokines in bronchoalveolar lavage fluid (BALF) from the CSE/PPE-induced chronic obstructibe pulmonary disease (COPD) model. (**a**) The timeline of mouse CSE/PPE sensitizaion and oral administration. Cell infiltration (**b**) was measured by Diff-quick stain methods (scale bar: 50 µm) and all images were taken at ×400 magnification. The total cells counts (**c**) were assessed by automatic cell counter. The number of macrophages (**d**) and neutrophils (**e**) was counted from microscopy images. The levels of IL-6 (**f**), MIP-2 (**g**) and MMP-9 (**n**) proteins were measured by ELISA and the levels of KC (**h**), MDC (**i**), eotaxin (**j**), RANTES (**k**), MCP-1 (**l**), and TARC (**m**) were analyzed by Q-plex. NETosis (**o**) was determined by Sytox Green fluorescence. Data are presented as means ± SD. The significant differences among the groups were analyzed using one-way analysis of variance by Bonferroni’s test (## *p* < 0.01 compared to the control group; * *p* < 0.05 and ** *p* < 0.01 compared to the CSE + LPS-stimulated group).

We investigated whether the oral administration of AGE attenuates lung inflammation in a COPD mouse model. The mice of each group were intranasally treated with CSE/PPE and orally administered with AGE and DEX from day 0 and day 7, respectively. The inflammatory cell count and pro-inflammatory factor levels were measured in the BALF extracted from each mouse group (Figure 4a). The effects of AGE on Diff-Quick staining of BALF cells, total number of cells, total macrophages, and total neutrophils are shown in Figure 4b–e. Compared to the BALF of the naïve group, the COPD group showed a markedly upregulated number of inflammatory cells, which was dose-dependently reduced in the AGE group.

Figure 4f–m shows the cytokine and chemokine assessments. The COPD group showed an increase in all factors, such as IL-6, MIP-2, KC, MDC, eotaxin, RANTES, MCP-1, and TARC, and these levels were downregulated in the AGE group. In addition, the increased MMP-9 and NETosis levels in the COPD group decreased as the AGE concentration increased. These results suggest that AGE may be able to ameliorate COPD by inhibiting the inflammatory responses.

### 3.4. Effect of AGE on Airway Inflammation, Alveolar Enlargement, Cytokines and Chemokines and NF-κB in Lung Tissue

**Figure 5:**
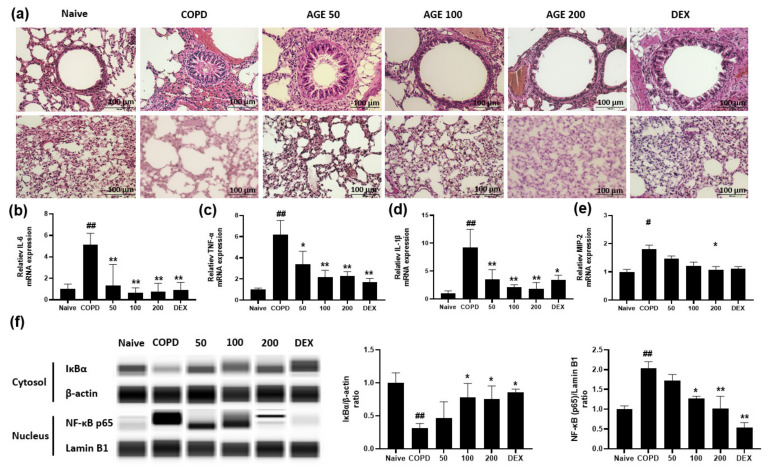
Effect of the *Artemisia gmelinii* ethanol extract (AGE) on airway inflammation and alveolar enlargement, cytokines and chemokines, and the activation of NF-κB pathways in lung tissues from the CSE (100%)/PPE (1.8 U/Head)-induced COPD mice. Following the schedule in Figure 4a, lung tissues from each group were collected and used to assess the airway inflammation and alveolar enlargement, cytokines and chemokines, and the activation of NF-κB pathways. The airway inflammation and alveolar enlargement (**a**) were measured by hematoxylin and eosin staining and all images were taken at × 200 magnification (scale bar: 100 µm). The mRNA levels of *IL6* (**b**), *TNFα* (**c**), *IL1β* (**d**), and *MIP2* (**e**) were measured by quantitative real-time polymerase chain reaction. IκBα, NF-κB p65, β-actin, and lamin B1 (**f**) were assessed and quantitative data were analyzed using Protein Simple capillary immunoassay (WES). Data are presented as means ± SEM. Significant differences among the groups were analyzed using one-way analysis of variance using Bonferroni’s test (# *p*  <  0.05 and ## *p* < 0.01 compared to the Naive group; * *p* < 0.05 and ** *p* < 0.01 compared to the COPD group) Naïve—Naive group; COPD—COPD group; 50—AGE 50 group; 100—AGE 100 group; 200—AGE 200 group; DEX—dexamethasone group.

From the lung tissues of each group, the histopathology, pro-inflammatory cytokines and chemokines and their mechanisms were investigated. The main features of COPD are airway inflammation, enlargement, and destruction leading to airflow limitation. As described in Figure 5a, the lung tissues of the Naive group did not display airway inflammation or destruction of alveolar walls, and the size of the alveolar space was normal. However, CSE and PPE contributed to the deterioration of airway inflammation and the destruction and enlargement of alveolar space, which were observed in the COPD group. In the AGE group, these effects were attenuated. We demonstrated that AGE attenuated airway inflammation and alveolar enlargement in lung tissues. Inflammation involves the response to pro-inflammatory cytokines and chemokines. Figure 5b–e shows mRNA levels of *IL6*, *TNFα*, *IL1β*, and *MIP2* in lung tissues. Similar to the in vitro results, these levels were increased in the COPD group and were markedly decreased in the AGE group. These results agreed with the protein levels of the cytokines and chemokines shown in Figure 4f–m and indicated that AGE suppresses the mRNA levels of pro-inflammatory cytokines and chemokines. As NF-κB signaling pathways are known to produce these proteins, the effects of AGE on the protein expression of IκBα and NF-κB p65 are shown in Figure 5f. The COPD group exhibited lower protein expression of IκBα in the cytosol, and NF-κB p65 in the nucleus was higher than that in the naïve group, but recovered in the AGE group. The levels of IκBα protein in the cytosol were markedly increased, and NF-κB p65 in the nucleus was significantly decreased in the AGE group. These results demonstrated that AGE can inhibit lung inflammation by suppressing the secretion of cytokines and chemokines via inhibiting NF-κB activation.

### 3.5. Active Compounds in AGE

**Figure 6:**
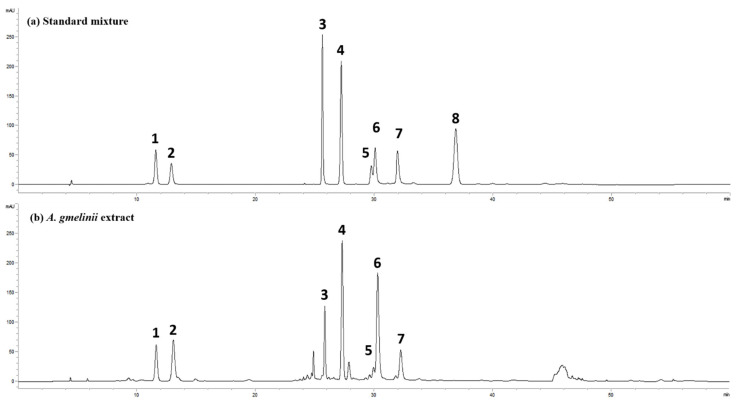
High-performance liquid chromatography profile of standard mixture (**a**) and *Artemisia gmelinii* extract (**b**): (1) scopolin; (2) chlorogenic acid; (3) hyperoside; (4) scopoletin; (5) 3,4-di-*O*-caffeoylquinic acid; (6) 3,5-di-*O*-caffeoylquinic acid; (7) 4,5-di-*O*-caffeoylquinic acid; (8) scoparone.

To investigate the active AGE components, we analyzed ACE using HPLC. The results showed seven main peaks which were identified as follows: (1), scopolin; (2), chlorogenic acid; (3), hyperoside; (4), scopoletin; (5), 3-4-di-*O*-caffeoylquinic acid; (6), 3-5-di-*O*-caffeoylquinic acid; (7), 4-5-di-*O*-caffeoylquinic acid (Figure 6). Scoparone used to discriminate between *A. capilliaris* and AG was not detected in AGE. In quantitative analysis, Table 1 shows that AGE included 8.6 mg/g of scolopin, 21.8 mg/g of chlorogenic acid 21.8 mg/g, 5.2 mg/g of hyperoside, 7.3 mg/g of scopoletin, 9.5 mg/g of 3-4-di-*O*-caffeoyl quinic acid, 34.6 mg/g of 3-5-di-*O*-caffeoyl quinic acid, and 5.2 mg/g of 4-5-di-*O*-caffeoyl quinic acid. A recent study showed that coumarins, such as scopoletin and scopolin, and phenolic compounds, such as chlorogenic acid and caffeoylquinic acid, are major constituents of *Artemisia* species, and possess various biological functions, including anti-inflammatory, anti-oxidative, anti-infection, anti-proliferation, and anti-diabetic effects [23,24]. Therefore, we examined whether the separated components (scopolin, chrologenic acid, hyperoside, scopoletin, 3-4-di-*O*-caffeoylquinic acid; (6), 3-5-di-*O*-caffeoylquinic acid; (7), 4-5-di-*O*-caffeoylquinic acid) altered CSE/LPS-induced IL-6 secretion in the MH-S cells. Most components, except scopoletin, suppressed IL-6 expression in a dose-dependent manner, as shown in Table 2. In particular, 3-5-di-*O*-caffeoyl quinic acid, which is the component with the highest concentration in AGE, had the best effect in inhibiting IL-6 production at a concentration of 10 ng/mL.

## 4. Discussion

Our study showed AGE as a good alternative to medications or supplements for lung health by suppressing CSE/PPE-induced lung inflammation in COPD mouse model and the inflammatory response via the inhibition of NF-κB and p38 in the CSE/LPS-stimulated MH-S alveolar macrophage cell line. AMs, which reside in the lumen of the airway, play an essential role in clearing harmful infectious particles that invade the respiratory tract, regulate innate alveolar defenses against respiratory infections, and present antigens to adaptive immune cells [25]. When AMs are stimulated and activated by foreign substances, such as CS and LPS, they begin to produce pro-inflammatory mediators, such as NO and prostaglandin E2 (PGE_2_), contributing to lung destruction [26,27]. iNOS and COX-2 produce NO and PGE_2_, respectively, which cause airway inflammation and modulate lung fibroblast functions [28,29]. Accordingly, a recent study reported that the mRNA expression of *NOS2* and *COX2* in lung tissues was increased in COPD patients compared to in non-smokers and smokers without COPD. Therefore, one of the therapeutic strategies for COPD is inhibiting iNOS. In this study, the mRNA and protein levels of *NOS2* and *COX2* were upregulated in CSE/LPS-stimulated AMs; however, in AGE-treated AMs, the mRNA and protein levels of *NOS2* and *COX2* were reduced, demonstrating that AG has anti-inflammatory properties (Figure 1). IL-6, TNF-α, IL-1β, and MIP-2 are representative pro-inflammatory cytokines and chemokines that are related to the pathogenesis of various lung diseases. IL-6 is related to systemic inflammation in COPD patients [30], and TNF-α and IL-1β induce fibroblast proliferation and enhance the synthesis of fibronectin and collagen during the progression of COPD [31]. Murine MIP-2, a functional homolog of human IL-8, is a chemokine that attracts neutrophils to the inflammation sites [32]. Our study showed that CSE/LPS caused increased mRNA levels of *IL6*, *TNFA*, *IL1B*, and *MIP2*. They were all suppressed in AGE-treated AMs (Figure 2a–d). From these results, the attenuating effects of AGE on lung damage may be related to the reduction in inflammation-related factors.

To determine which mechanism is related to the suppression of pro-inflammatory factors, the MAPK/NF-κB signaling pathways, which are involved in the secretion of IL-6, TNF-α, IL-1β, and MIP-2, as well as iNOS and COX-2 were measured. Activation of the MAPK/NF-κB signaling pathway is related to pulmonary inflammation induced by CS. Previous studies have shown that toxic substances in CS, such as tar, nicotine, acrolein, and H_2_O_2_, activate the MAPK/NF-κB signaling pathway, leading to lung injury and emphysema [33,34]. In the present study, CSE/LPS increased the phosphorylation of all MAPK members, while phosphorylation of p38 was inhibited most effectively in AGE-treated AMs. p38 is involved in fibrosis, neutrophilia, apoptosis, and cytokine production during lung injury. In AMs, as well as in the alveolar walls of smokers with COPD, the phosphorylation of p38 was increased, which was associated with lung dysfunction [32]. This study showed that AGE acted as a p38 inhibitor, and, through the p38 pathway, suppressed the secretion of pro-inflammatory mediators (Figure 2e). Western blot data also showed that the NF-κB signaling pathway was activated in CSE/LPS-stimulated AMs, possibly by degrading IκBα in the cytosol and translocating NF-κB p65 into the nucleus. However, AGE inhibited this reaction. In addition, from fluorescence confocal microscopy data, deactivation of the NF-κB signaling pathway was observed in AGE-treated AMs (Figure 2f and Figure 3a,b). These results indicated that AGE inhibited the secretion of pro-inflammatory cytokines and chemokines via suppression of NF-κB p65 nuclear translocation and phosphorylation of p38 in the MAPK family.

Meanwhile, accumulated evidence indicates that the Nuclear factor-E2-related factor 2 (Nrf2)/heme oxygenase-1 (HO-1) pathway, a major antioxidant defense system, regulates inflammatory responses by suppressing the excessive production of ROS, inflammatory mediates, and pro-inflammatory cytokines [35]. In addition, recent studies have reported that the inhibition of NF-κB of phytochemicals is related to the activation of Nrf2/HO-1 [36]. Accordingly, to investigate whether the anti-inflammatory effects of AGE is related to the activation of Nrf2/HO-1 signaling pathways, we analyzed the expression of Nrf2 and HO-1 following AGE treatment in the CSE/LPS-stimulated MH-S. As for the results of the Western blot analysis, AGE increased the expression of HO-1 by further promoting nuclear translocation of Nrf2 in a dose-dependent manner (Figure S1). From these observations, we hypothesized that AGE may regulate the CSE/LPS-induced inflammatory response by its antioxidant properties as well as anti-inflammatory properties; however, further studies are needed to determine the molecular mechanisms associated with Nrf2/HO-1.

Based on our in vitro experiments, we examined the protective effect of AGE on lung damage using the CSE/PPE mouse model, in which COPD is induced and demonstrates emphysema and inflammation [37,38]. Numerous studies have shown that inflammatory cell counts are increased in the BALF of CSE- or PPE-treated mouse models [39,40]. In COPD patients, the number of AMs and neutrophils is markedly increased in the BALF, airway, and sputum, indicating a correlation between the number of AMs and COPD severity [41]. Therefore, the total inflammatory cell count is a critical biomarker for confirming the diagnosis of COPD. In our study, total cell, macrophage, and neutrophil counts were increased in the BALF of the COPD group, which was treated with CSE/PPE, compared to the Naïve group. In the AGE group, which was orally administered 50, 100, and 200 mg/kg of AGE, these levels were reduced. Consistent with these results, the Diff-quick stain method was used to measure cell infiltration, and revealed a decrease in macrophages and neutrophils in the AGE-treated group (Figure 4b–e). The results revealed that AGE administration clearly reduced the unbridled infiltration of total cells, macrophages, and neutrophils, in BALF.

Persistent infiltration of immune cells enhances the release of inflammatory cytokines and chemokines. Heightened cytokine and chemokine secretion is one of the main factors in the exacerbation of COPD. MIP-2 and KC are pro-inflammatory chemokines known to recruit neutrophils in the lungs [32]. Eotaxin is a chemokine that plays a role in recruiting eosinophils, causing airway inflammation in COPD [42]. The other chemokine, RANTES, increases the number of eosinophils and T cells in the large airways of COPD patients. MCP-1 is also expressed in the airways of patients with COPD, recruiting and activating inflammatory cells [43,44]. TARC and MDC function as chemoattractants for Th2 cells, and are related to a decline in forced expiratory volume in 1 s (FEV_1_) in COPD patients [45,46]. These proteins are also related to hypertrophy and hyperplasia of airway smooth muscle, resulting in irreversible airway obstruction. In this study, the BALF of the COPD group showed a significant increase in all these factors, whereas these levels were decreased in the AGE group (Figure 4f–m).

MMP-9, a 92-kDa type IV collagenase/gelatinase B family member of the MMP family, is secreted by neutrophils and macrophages. Under basal conditions, MMP-9 levels are generally low, but overproduction is related to alveolar destruction and emphysema, leading to various lung diseases, such as lung cancer, asthma, fibrosis, and interstitial lung diseases [47]. In clinical trials, serum MMP-9 levels were markedly higher in patients with COPD than in the non-smoker control group, and an increase in MMP-9 was associated with a decrease in FEV_1_% [48]. In our study, the level of MMP-9 in the COPD group was significantly upregulated compared to that in the Naïve group, and the level was significantly decreased in the AGE group (Figure 4n), which contributed to the attenuation of alveolar destruction (Figure 5a).

Proteolytic enzymes secreted through NETosis, the formation of NETs accompanied by the death of neutrophils, and inflammatory mediators secreted by AMs cause airway inflammation, airway wall thickening, and the destruction of alveolar space observed in COPD, which result in irreversible airflow obstruction, [49]. In our study, CSE/PPE induced an increase in NETosis (Figure 4o), MMP-9, IL-6, TNF-α, IL-1β, MIP-2, KC, RANTES, MCP-1, MDC, TARC, and eotaxin levels in the BALF, inflammatory cell infiltration, luminal occlusion by secretion of mucous glycoproteins and inflammatory exudates, airway wall thickness, mucus hypersecretion, alveolar enlargement, and alveolar destruction (Figure 5a), as observed in the lung tissues of the COPD group. These lung injuries were alleviated in a dose-dependent manner in the lung tissues of the AGE group. Moreover, mRNA levels of IL-6, TNF-α, IL-1β, and MIP-2 were increased in the lung tissues of the COPD group and were decreased in the AGE group (Figure 5b–e). Similar to in vitro studies, we examined whether AGE suppressed the activation of the NF-κB signaling pathway in the lung tissue and observed the inhibition of translocating NF-κB p65 into the nucleus in the AGE group (Figure 5f). These data suggested that AGEs could attenuate lung inflammation by suppressing the NF-κB signaling pathway.

*Artemisia* plants, including AG, have been used as medicinal herbs for the treatment of colds, coughs, headaches, dyspepsia, and colic in Europe, Asia, and North America, because the genus *Artemisia* contains mainly terpenoids, flavonoids, coumarins, caffeoylquinic acids, sterols, and acetylenes. Accordingly, we identified the components of AGE by quantitative HPLC analysis to identify the main active compounds possessing anti-inflammatory effects. From the HPLC profile of AGE, we identified a variety of active compounds, such as scopolin, chlorogenic acid, hyperoside, scopoletin, 3,4-di-*O*-caffeoylquinic acid, 3,5-di-*O*-caffeoylquinic acid, and 4,5-di-*O*-caffeoylquinic acid. There have been many reports about the anti-oxidant and anti-inflammatory activities of these compounds: scopolin and scopolectin isolated from *A. iwayomogi* suppresses the production of ROS in RAW 264.7 cells [50]; chlorogenic acid suppresses allergic inflammatory responses via the inhibition of STAT-6 and JNK activation [51]; caffeoylquinic acid reduces pro-inflammatory cytokines, such as TNF-α and IL-1β, and attenuates oedema volume in rat [52]; and caffeoylquinic acid-rich fraction purified from *A. absinthium* L. and *A. ludoviviana* Nutt suppresses ROS production [53]. Based on these reports, we measured IL-6 in the CSE/LPS-stimulated MH-S cells to evaluate the anti-inflammatory activities of these compounds. Consistent with these studies, scopolin, chlorogenic acid, hyperoside, 3,4-di-*O*-caffeoylquinic acid, 3,5-di-*O*-caffeoylquinic acid, and 4,5-di-*O*-caffeoylquinic acid suppressed the IL-6 increase induced by CSE/LPS stimulation in our study. In particular, 3,5-di-*O*-caffeoylquinic acid, the predominant compound in AGE, effectively decreased IL-6 expression at a low concentration of 10 ng/mL. Given the results, various components, especially 3,5-di-*O*-caffeoylquinic acid, in AGE contribute to the anti-inflammatory characteristics of AGE. We anticipated that the synergistic effects of these compounds will not be limited in lung disease and will adapt to other diseases.

The studies on the pharmacokinetics and bioavailability of AGE have not been reported, despite the use of AGE in traditional medicine which has existed for a long time. Fortunately, recently several studies have reported the pharmacological and biological effects of chlorogenic acid, one of the functional components of AGE [54]. Chlorogenic acid has been known to have an improving effect in various animal models of respiratory diseases such as ALI, lung fibrosis, pneumonia, and lung cancer model [55,56,57]. It is possible that chlorogenic acid directly affects lung-related inflammation, as when chlorogenic acid-rich plant extract was orally administered, the highest content of chlorogenic acid was detected in plasma after 30 min [58]. Subsequently, a large amount of chlorogenic acid was detected in the liver, lung, heart, spleen, and kidney. Given the similarity of their chemical structures, it may be possible that various components of AGE, including caffeoylquinic acid, also sufficiently reached the blood and lung and then directly affected immune cells such as alveolar macrophages. In future studies, we will investigate the efficacy and organ distribution of functional components of AGE in various animal models of pulmonary diseases.

Meanwhile, in terms of the usage of dietary supplements, functional foods, and pharmaceuticals for lung health, the safety of natural products is as important as their effectiveness. Although the safety of AG has not been reported, there is accumulated evidence supporting the safety and efficacy of caffeoylquinic acid, chlorogenic acid, and natural coumarins such as scopoletin, the main compounds of AGE, in pre-clinical, clinical, and toxicity studies [59,60,61]. In addition, a recent study reported that the intake of *A. capillaris*, with a similar chemical profile to AG, improved disease severity without side effects in a clinical trial for patients with liver diseases [62]. These and our findings suggest that AGE could be an effectual alternative to medications with side effects such as weight loss, headaches, and insomnia, in the prevention and treatment of inflammatory lung disease.

## 5. Conclusions

In conclusion, AGE inhibited the secretion of pro-inflammatory cytokines, chemokines, iNOS, and COX-2, by inactivating the MAPK/NF-κB signaling pathways in CSE/LPS-stimulated MH-S cells. Oral administration of AGE attenuated clinical symptoms, including the infiltration of inflammatory cells such as macrophages and neutrophils, and increased pro-inflammatory cytokines and chemokines in the BALF obtained from CSE/PPE-induced COPD mice. Furthermore, the attenuation of alveolar enlargement, mucus hypersecretion, bronchiole thickness, and increased pro-inflammatory cytokines associated with NF-κB signaling pathways were observed in the lungs of AGE-treated mice. These attenuating effects are associated with scopolin, chlorogenic acid, hyperoside, 3,4-di-*O*-caffeoylquinic acid, 3,5-di-*O*-caffeoylquinic acid, and 4,5-di-*O*-caffeoylquinic acid, which are the main components in AGE. Considering both the in vitro and in vivo results, we suggest that AGE may have beneficial effects on lung diseases including COPD. However, further studies are needed to determine the mechanisms underlying the inflammatory cell infiltration and proteolytic enzyme function in the development of COPD.

## Figures and Tables

**Table 1:** The quantification of seven components in *Artemisia gmelinii* extract.

Compound	Regression Equation	Regression Coefficient (R^2^)	Content (mg/g)
Scopolin	y = 8.1105x − 3.6018	0.9999	8.6
Chlorogenic acid	y = 5.3291x − 42.167	1.0000	21.8
Hyperoside	y = 17.799x + 3.6018	1.0000	5.2
Scopoletin	y = 22.339x − 194.46	0.9990	7.3
4,5-di-*O*-caffeoylquinic acid	y = 3.8996x + 10.436	0.9996	5.2
3,5-di-*O*-caffeoylquinic acid	y = 7.2616x + 35.623	0.9998	34.6
3,4-di-*O*-caffeoylquinic acid	y = 8.2834x + 16.766	1.0000	9.5
Scoparone	y = 24.082x − 114.77	0.9994	ND

**Table 2:** The inhibitory effects of scopolin, chrologenic acid, hyperoside, scopoletin, 3-4-di-*O*-caffeoylquinic acid, 3-5-di-*O*-caffeoylquinic acid, 4-5-di-*O*-caffeoylquinic acid on IL-6 production by CSE/LPS-stimulated MH-S cells.

Identified Components	Il-6 (ρg/mL)				
	Naive	CSE/LPS	10 ng/mL	100 ng/mL	1000 ng/mL
Scopolin	784.38 ± 50.39	7029.17 ± 1214.07 ^##^	5340.63 ± 726.96 **	5050.00 ± 641.53 **	5286.75 ± 753.36 **
Chlorogenic acid	762.50 ± 75.70	11,010.42 ± 1582.40 ^##^	12,840.63 ± 762.50	8128.13 ± 590.90 **	8000.00 ± 718.10 **
Hyperoside	762.50 ± 75.70	11,010.42 ± 1582.40 ^##^	7237.50 ± 1209.01 **	6406.25 ± 1620.76 **	4078.13 ± 318.42 **
Scopoletin	784.38 ± 50.39	7029.17 ± 1214.07 ^##^	8515.63 ± 1092.70	6895.83 ± 1087.02	6641.67 ± 466.43
4,5-di-*O*-caffeoylquinic acid	784.38 ± 50.39	7029.17 ± 1214.07 ^##^	8090.63 ± 593.58	5537.50 ± 97.36 *	4725.00 ± 234.30 **
3,5-di-*O*-caffeoylquinic acid	762.50 ± 75.70	11,010.42 ± 1582.40 ^##^	5346.88 ± 726.96 **	5122.92 ± 697.35 *	4214.58 ± 750.66 **
3,4-di-*O*-caffeoylquinic acid	762.50 ± 75.70	11,010.42 ± 1582.40 ^##^	11,971.88 ± 210.50	8343.75 ± 696.90 **	6685.42 ± 914.50 **

Significant differences among the groups were analyzed using one-way analysis of variance by Bonferroni’s test ## *p* < 0.01 compared to the Naive group; * *p* < 0.05 and ** *p* < 0.01 compared to the CSE/LPS group).

## Data Availability

The data presented in this study are available upon request to the corresponding author.

## Supplementary Materials

The following supporting information can be downloaded at: https://www.mdpi.com/article/10.3390/antiox11030568/s1, Figure S1: Effect of the *Artemisia gmelinii* ethanol extract (AGE) on the activation of Nrf2/HO-1 pathways in the MH-S macrophages.

## Abbreviations

The following abbreviations are used in this manuscript.

AG	*Artemisia gmelinii*
AGE	*Artemisia gmelinii* ethanol extract
AM	Alveolar macrophage
BALF	Bronchoalveolar lavage fluid
COPD	Chronic obstructive pulmonary disease
COX-2	Cyclooxygenase-2
CS	Cigarette smoke
CSE	Cigarette smoke extract
ELISA	Enzyme-linked immunosorbent assay
HO-1	Heme oxygenase-1
HPLC	High-performance liquid chromatography
IL-6	Interleukin-6
iNOS	Inducible nitric oxide synthase
IκBα	Nuclear factor of kappa light polypeptide gene enhancer in B-cells inhibitor, alpha
KC	Keratinocytes-derived chemokine
LPS	Lipopolysaccharide
MAPK	Mitogen-activated protein kinase
MCP-1	Monocyte chemoattractant protein-1
MDC	Macrophage-derived chemokine
MIP-2	Macrophage inflammatory protein-2
MMP-9	Matrix metalloproteinase-9
NET	Neutrophil extracellular traps
NF-κB	Nuclear factor kappa-light-chain-enhancer of activated B cells
NO	Nitric oxide
Nrf2	Nuclear factor-E2—related factor 2
PDTC	Ammonium pyrrolidine dithiocarbamate
PPE	Porcine pancreas elastase
RANTES	Regulated upon Activation, Normal T Cell Expressed and Presumably Secreted
ROS	Reactive oxygen species
TARC	Thymus and activation-regulated chemkine
TNF-α	Tumor necrosis factor-α
