# Supplementary material for: Artemisia gmelinii Attenuates Lung Inflammation by Suppressing the NF-κB/MAPK Pathway
Source: Antioxidants (Basel). 2022 Mar 16;11(3):568. doi: 10.3390/antiox11030568 (PMC8944700; doi:10.3390/antiox11030568)
Supplement: Supplementary file 1 [file antioxidants-11-00568-s001.zip › antioxidants-1595491-supplementary.pdf]

Supplementary data

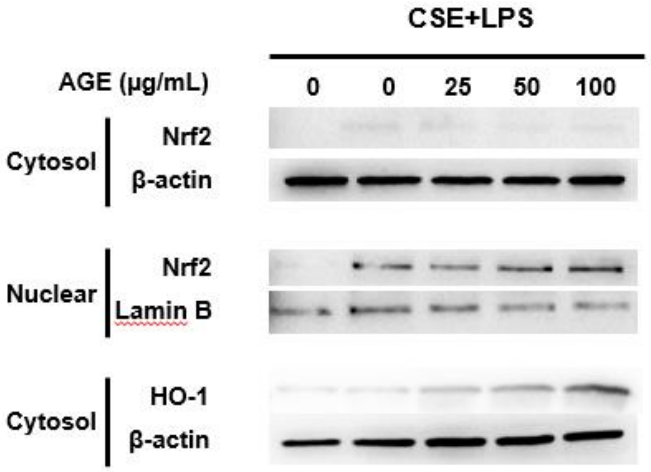

Figure S1. Effect of the *Artemisia gmelinii* ethanol extract (AGE) on the activation of Nrf2/HO-1 pathways in the MH-S macrophages. To determine the activation of Nrf2/HO-1 pathways, M H-S macrophages were pre-treated with 25, 50, 100 μg/mL of AGE for 2 h and then treated with CSE (1%)/LPS (10 ng/mL) for 2 h (Nrf2) and 12h (HO-1), respectively. Protein expression of Nrf2, HO-1, β-actin, and lamin B1 was assessed by western blotting. MH-S macrophages w ere collected in DPBS and lysed in an ice-cold cell lysis buffer with protease inhibitor to extra ct protein. The concentration of extracted proteins in each sample was measured using the Bra dford protein assay. Forty micrograms of the protein mixture from each sample was loaded in to the wells of Mini-PROTEAN® TGX™ Precast Gels. After electrophoresis, the proteins in the gels were transferred onto western blotting filter membranes for 50 min at 25 V, using a Pie rce G2 Fast Blotter (Thermo Fisher Scientific). After the transfer, membranes were blocked wit h 5% skim milk in PBS-Tween 20 (PBST) for 30 min at room temperature. Then, the membra nes were incubated with diluted Nrf2, HO-1, β-actin, and lamin B1 antibodies overnight at 4℃. The next day, the membranes were washed three times with PBST for 5 min. After wash ing, the membranes were incubated with anti-mouse or rabbit IgG antibodies conjugated with HRP for 1 h at room temperature and washed three times with PBST for 5 min. Protein band s were quantified using a Fusion Solo S (Vilber, Lamirault, France).
